# Cucurbituril Ameliorates Liver Damage Induced by Microcystis aeruginosa in a Mouse Model

**DOI:** 10.3389/fchem.2021.660927

**Published:** 2021-04-14

**Authors:** Na'il Saleh, Saad Al-Jassabi, Ali H. Eid, Werner M. Nau

**Affiliations:** ^1^Department of Chemistry, College of Science, United Arab Emirates (UAE) University, Al Ain, United Arab Emirates; ^2^Faculty of Medicine, Unishams University, Kuala Ketil, Malaysia; ^3^Department of Basic Medical Sciences, College of Medicine, QU Health, Qatar University, Doha, Qatar; ^4^Biomedical and Pharmaceutical Research Unit, QU Health, Qatar University, Doha, Qatar; ^5^School of Engineering and Science, Jacobs University Bremen, Bremen, Germany

**Keywords:** cyanobacterial crude extract, chemoprotectant, cucurbituril, *Microcystis aeruginosa*, liver damage

## Abstract

*Microcystis aeruginosa* is a cyanobacterium that produces a variety of cyclic heptapeptide toxins in freshwater. The protective effects of the macromolecular container cucurbit[7]uril (CB7) were evaluated using mouse models of cyanotoxin-induced liver damage. Biochemical analysis of liver function was performed to gauge the extent of liver damage after exposure to cyanobacterial crude extract [CCE; LD_50_ = 35 mg/kg body weight; intraperitoneal (i.p.)] in the absence or presence of CB7 (35 mg/kg body weight, i.p.). CCE injection resulted in liver enlargement, potentiated the activities of alanine aminotransferase (ALT) and glutathione S-transferase (GST), increased lipid peroxidation (LPO), and reduced protein phosphatase 1 (PP1) activity. CCE-induced liver enlargement, ALT and GST activities, and LPO were significantly reduced when CB7 was coadministered. Moreover, the CCE-induced decline of PP1 activity was also ameliorated in the presence of CB7. Treatment with CB7 alone did not affect liver function, which exhibited a dose tolerance of 100 mg/kg body wt. Overall, our results illustrated that the addition of CB7 significantly reduced CCE-induced hepatotoxicity (*P* < 0.05).

## Introduction

**Figure 1:**
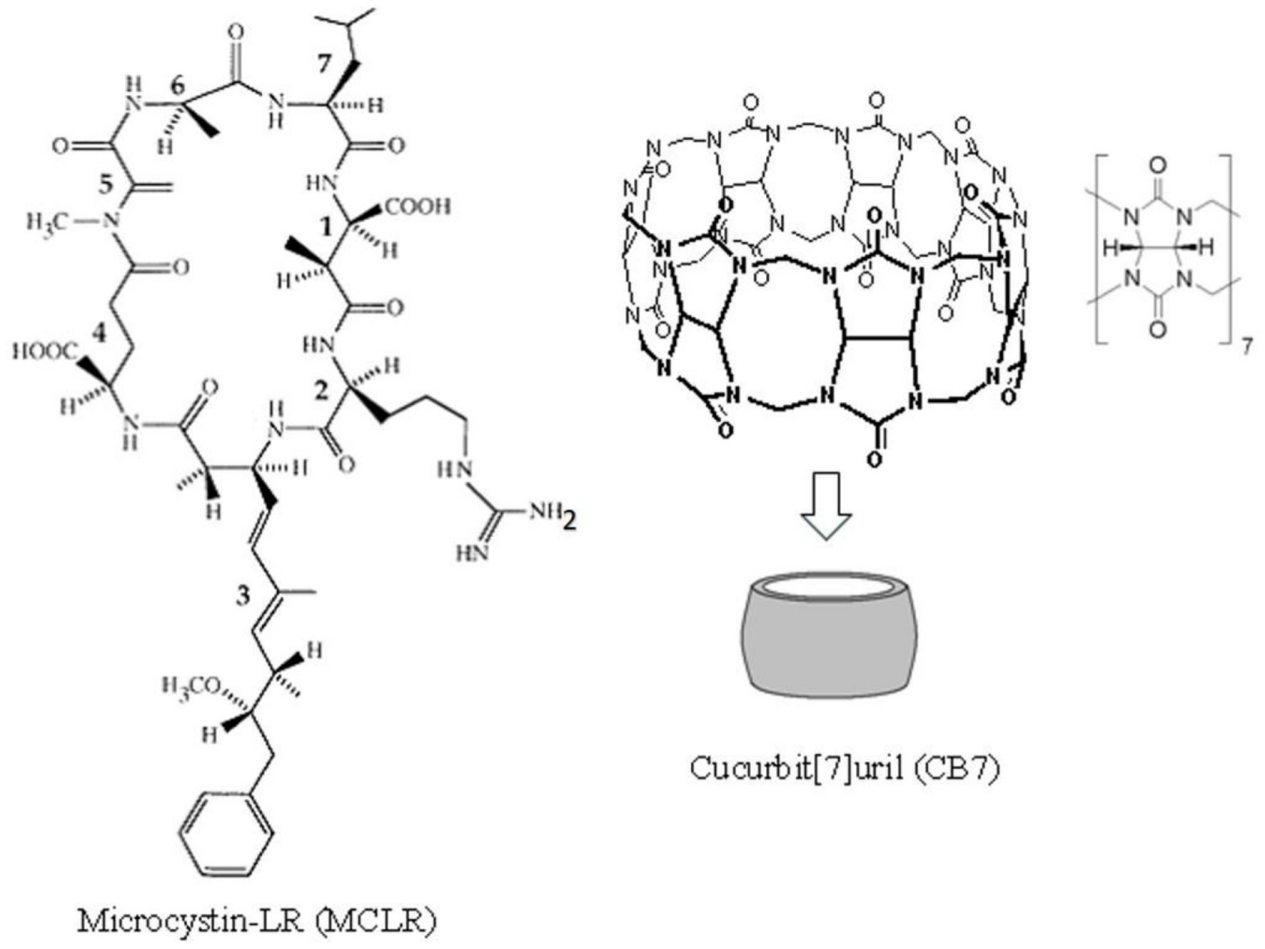
The chemical structures for cucurbit[7]uril (CB7) and microcystin-LR (MC-LR). MC-LR is a cyclic heptapeptide toxin comprising (1) methylaspartic acid; (2) l-arginine; (3) 3-amino-9-methoxy-2,6,8-trimethyl-10-phenyldeca-4,6-dienoic acid; (4) d-glutamic acid; (5) N-methyldehydro-alanine; (6) d-alanine; and (7) l-leucine.

The use of synthetic macromolecules for biological and medicinal applications has always been a popular approach (Yin et al., 2021). Accordingly, several studies have demonstrated the non-toxic and biocompatible nature of synthetic cucurbiturils (CBs) (Uzunova et al., 2010; Zhang et al., 2018). CBs are molecular containers comprising two hydrophilic carbonyl-lined portals with a central hydrophobic cavity. They have been synthesized in different sizes (Figure 1 for cucurbit[7]uril [CB7]) (Lee et al., 2003). Because of these properties, the bioactivity of the CB7 host–guest complexes (Cheng et al., 2018) and their clinical applications *in vitro* and *in vivo* have been confirmed by several studies. For example, a previous study revealed that CB significantly decreased the hepatotoxicity caused by nitidine chloride (Li W. et al., 2017) as well as trazodone and its metabolite *m*-chlorophenylpiperazine (Huang et al., 2018a). Further, the antidotal activity of CB7 against paraquat, a toxic pesticide, was observed both *in vitro* and *in vivo* (Zhang et al., 2019a,b). Another study reported that a high affinity between CB7 and sorafenib, an anticancer drug (Yang et al., 2017), and bedaquiline (Kuok et al., 2018) and clofazimine (Li et al., 2016), antituberculosis drugs, enabled the host to decrease the cardiotoxicity of these drugs without affecting their activity *in vitro* and *in vivo* in zebrafish models (Yang et al., 2017). In the same year, it was reported that CB reduced the neurotoxicity of pentylenetetrazol in zebrafish and mouse models (Huang et al., 2018b). Additionally, acyclic CB derivatives were shown to have antidotal activity against methamphetamine in rats (Ganapati et al., 2017).

Although several macrocyclic hosts have been used to reverse the effects of poisonous substances (Yin et al., 2021), CB7 as an antidote to the toxicity of cyanobacterial crude extract (CCE) has been validated in in *vivo* studies. Cyanobacteria produce a wide range of cyclic peptide hepatotoxins, such as microcystins and nodularin. These secondary metabolites of cyanobacteria can be detected both in freshwater and marine environments. Figure 1 presents the chemical structure of microcystin-LR (MC-LR), a predominant toxin in CCE, as an example (Dittmann and Wiegand, 2006). Cyanobacteria are responsible for wildlife fatalities and adverse health effects in humans in countries where drinking water supplies are contaminated with these microorganisms (Al-Jassabi, 2005). Alarmingly, some of these cyanobacterial toxins are powerful promoters of liver tumor and potent inhibitors of protein phosphatase 1 (PP1) and PP2A catalytic subunits (PP1c/PP2Ac) that are critical for cytoskeletal integrity (Goldberg et al., 1995). During cyanobacterial bloom lysis in aquatic ecosystems, a mixture of toxins and other cyanobacterial and bacterial components are released in the water, which affects various aquatic organisms.

In this study, we analyzed the effects of CCE on liver function in mice with and without CB7 co-administration. Previous studies reported that the maximum tolerated dosage of CB7 is 250 mg/kg in mice, which is at least three-fold less than its *LD*_50_ (Uzunova et al., 2010). The LD_50_ of CCE in this study was 35 mg/kg body weight intraperitoneally (i.p.). International guidelines permit the analysis of doses at least 20-fold less than the LD_50_ of CB7 to evaluate its protective activity, which justifies the use of CB7 as an antidote agent at a dose of 35 mg/kg body weight.

## Materials and Methods

### Chemicals

All chemicals were purchased from Sigma-Aldrich. To calculate the CB7 concentrations, the water content in the bottle was assumed to be 20%, as stated by the supplier.

### Microcystis Cells

Different sites of the King Talal reservoir, Jordan, were targeted as sources of *Microcystis aeruginosa* during summer (May to October). We followed previously reported methods for isolating the cells and culturing them in medium (Dittmann and Wiegand, 2006). During cultivation, freeze-dried cells were used to extract cyanobacterial toxins using the method introduced by Mazur and Plinski (2003). To determine the LD_50_ of the extracted toxin, we followed the method reported by (Fawell et al., 1999).

### Animal Treatment and Blood Sample Collection

We used 5–7-week-old male BALB/c mice (average weight, 30 g). Mice were obtained from the animal house unit of the Yarmouk University and were housed in stainless metal cages, with *ad libitum* access to food and tap water for the duration of the study. The animals were maintained on a 12/12-h light–dark cycle at a temperature of 23–26°C. The Animal Care and Use Committee at Yarmouk University approved the study procedure.

The mice were divided into four groups of 10 animals each. Group 1 (control) received only a single 0.5-mL i.p. dose of physiologic saline (neutral pH); group 2 received CB7 injection (35 mg*/*kg); group 3 received free toxin CCE (35 mg*/*kg), and group 4 received both the toxin and CB7 (35 mg/kg each). Mice were sacrificed after 24 h. A second set of experiments (groups 5–8) was performed in which the mice received i.p. injections of CB7 aqueous solutions (2–100 mg/kg) at physiological pH (10 mice for each concentration).

To assess the activities of alanine aminotransferase (ALT) and glutathione S-transferase (GST), blood was collected from the animals and the serum was stored at −70°C. The excess blood was then removed by perfusing the liver with Hanks' balanced salt solution. The IKA Ultra-Turax homogenizer was used to homogenize liver tissues in phosphate-buffered saline (pH 7.2). The supernatant in the centrifuged homogenate was stored at −70°C to measure PP1 activity and lipid peroxidation (LPO).

### ALT Assay

To measure ALT activity in the serum samples, we followed the method outlined previously by Gehringer et al. (2003).

### GST Assay

GST (EC 2.5.1.18) activity was measured in the liver homogenate using 1-chloro-2,4-dinitrobenzene as a substrate according to the method outlined by Habig et al. (1974).

### LPO Assay

The thiobarbituric acid (TBA) method recommended by Hosseinzadeh et al. (2007) was used to assess LPO.

### PP1 Activity Assay

To determine the activity of PP1, the kinetics of the generation of a yellow product from the dephosphorylation of *para*-nitrophenyl phosphate was estimated using a spectrophotometer, as described by Yuan et al. (2006).

### Statistical Analysis

All results were expressed as mean ± standard error of the mean (SEM) for 10 mice per group. One-way analysis of variance followed by Tukey's *post-hoc* test was used to determine the significance of differences between the groups. Statistical significance was indicated by a *P* of ≤ 0.05. All statistical analyses were performed using SigmaStat statistical software (version 3.5).

## Results

**Figure 2:**
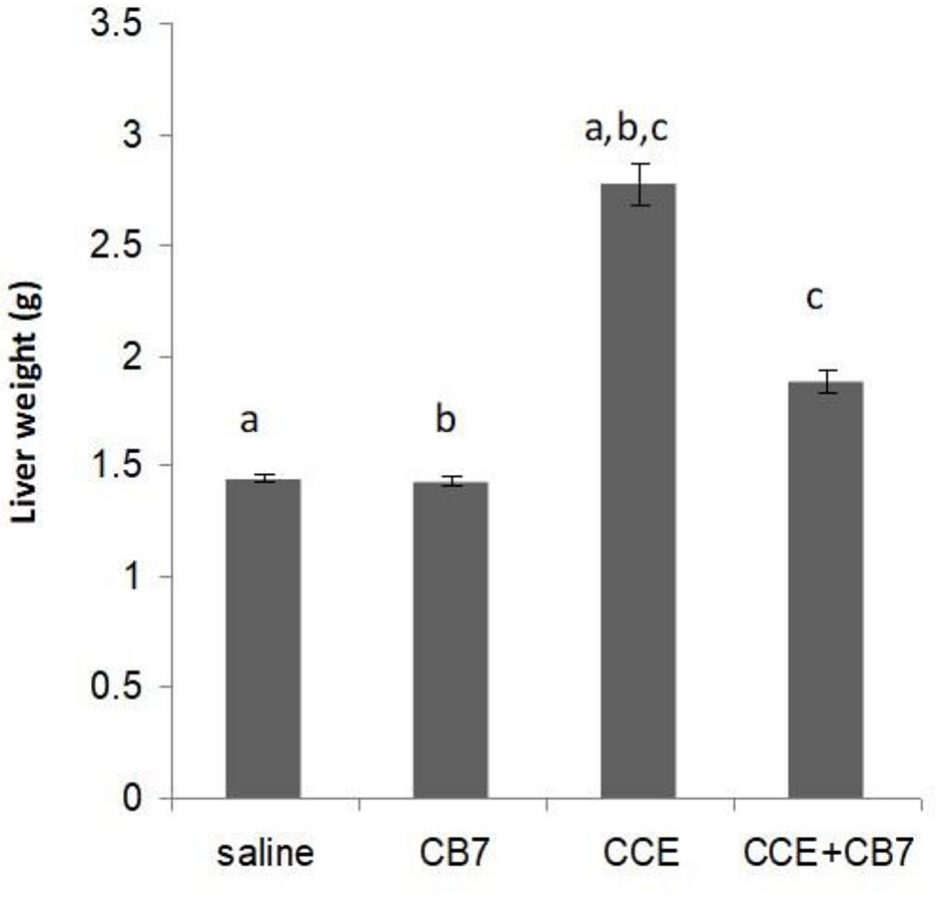
Effects of cucurbit[7]uril (CB7) on cyanobacterial crude extract (CCE)-induced liver damage. Mice were divided into groups 1–4 and the livers from mice in each group were weighed. Data are presented as mean ± SEM. Bars with similar letters represent statistically significant differences. Ten mice per group (*n* = 10).

Twenty-four hours after the i.p. injection, a significant increase in the liver mass was observed in group 3 (Figure 2 and Table 1) compared with group 1 (1.44 vs. 2.77 g; *P* < 0.05). This increase was attributable to massive intrahepatic hemorrhage and the pooling of blood in the liver (data not shown). CB7 alone (Table 2) did not significantly affect the liver mass (*P* > 0.05). However, co-administration of CB7 and CCE significantly nullified the increase in liver mass that was seen in group 3 (1.88 vs. 2.77 g; *P* < 0.05), which implies a protective effect of CB7 on CCE-induced liver enlargement.

**Table 1:** Effects of microcystin-LR on the liver function of BALB/c mice in the presence and absence of cucurbit[7]uril (CB7); 10 mice per group (*n* = 10).

**Groups**	**Liver weight (g)**	**ALT (U/L)**	**GST (U/L)**	**LPO (μM)**	**PP1 (U/mg)**
Group 1 (Normal)	1.44 ± 0.02	550 ± 5	2.57 ± 0.07	0.067 ± 0.01	0.880 ± 0.01
Group 2 (CB7)	1.43 ± 0.02	620 ± 5	2.59 ± 0.06	0.072 ± 0.01	0.566 ± 0.01
Group 3 (Toxin)	2.77 ± 0.09	1950 ± 51	12.22 ± 0.33	2.152 ± 0.04	0.165 ± 0.002
Group 4 (Toxin + CB7)	1.88 ± 0.05	1485 ± 39	10.34 ± 0.34	1.877 ± 0.04	0.311 ± 0.007

*ALT, alanine aminotransferase; GST, glutathione S-transferase; LPO, lipid peroxidation; PP1, protein phosphatase-1*.

**Table 2:** Effects of various concentrations of cucurbit[7]uril (CB7) on liver function in BALB/c mice; 10 mice per group (*n* = 10).

**Groups**	**Liver weight (g)**	**ALT (U/L)**	**GST (U/L)**	**LPO (μM)**	**PP1 (U/mg)**
Group 5 (2 mg/kg)	1.41 ± 0.02	575 ± 5	2.55 ± 0.06	0.071 ± 0.01	0.583 ± 0.01
Group 6 (10 mg/kg)	1.47 ± 0.03	545 ± 5	2.55 ± 0.06	0.069 ± 0.01	0.660 ± 0.01
Group 7 (40 mg/kg)	1.42 ± 0.02	562 ± 6	2.59 ± 0.07	0.078 ± 0.01	0.638 ± 0.02
Group 8 (100 mg/kg)	1.44 ± 0.03	628 ± 7	2.57 ± 0.06	0.097 ± 0.02	0.648 ± 0.02

*ALT, alanine aminotransferase; GST, glutathione S-transferase; LPO, lipid peroxidation; PP1, protein phosphatase-1*.

**Figure 3:**
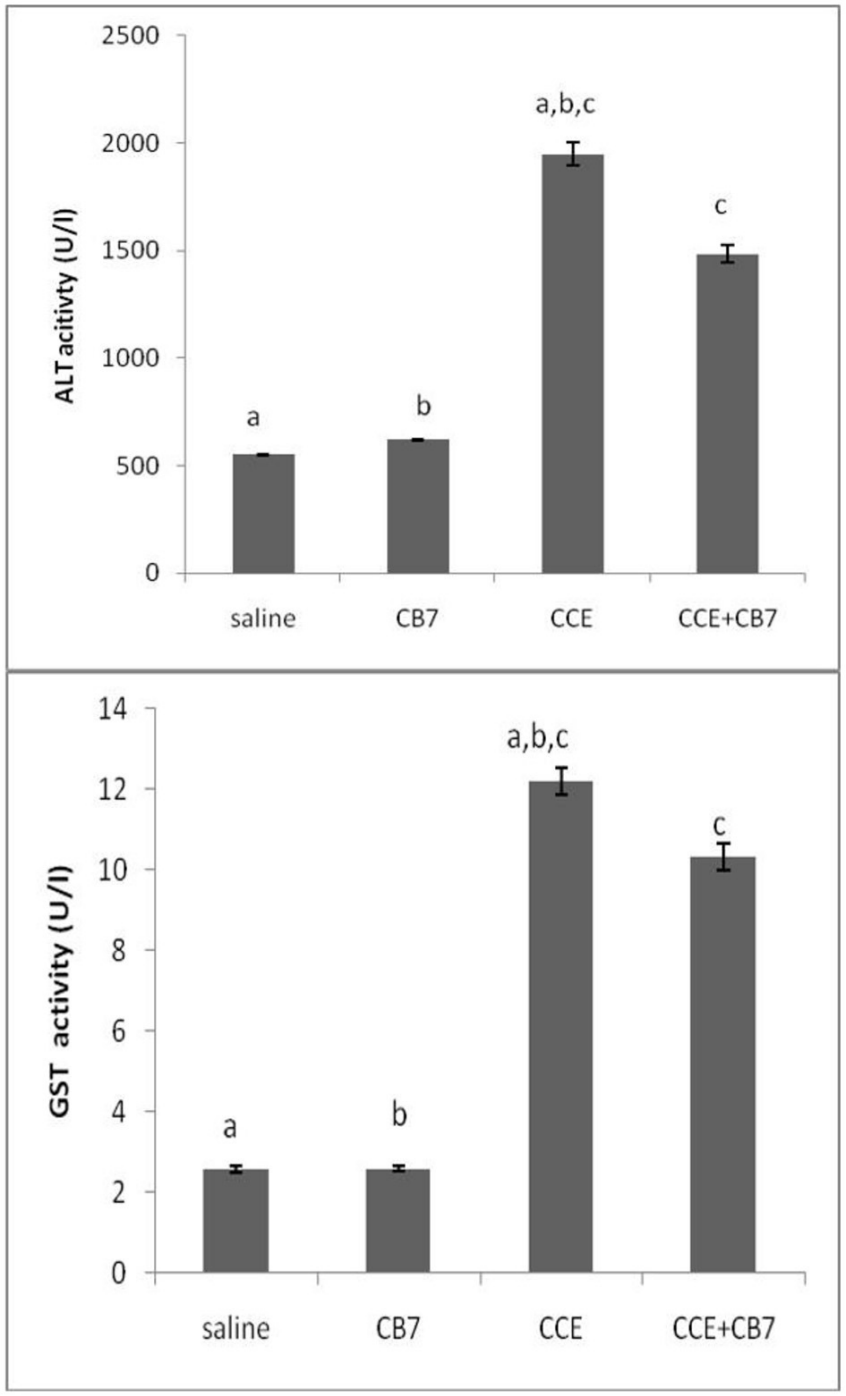
**(A)** The activity of alanine aminotransferase (ALT) and **(B)** glutathione S-transferase (GST) in the livers of mice in each group. Data are presented as mean ± SEM. Bars with similar letters represent statistically significant differences. Ten mice per group (*n* = 10).

The effects of CCE on various biochemical markers of hepatic function were also determined in the presence and absence of CB7 (Figure 3A and Table 1). Group 2 did not exhibit a significant increase in ALT activity; however, group 3 exhibited a robust increase in ALT activity compared with that in the control group 1 (1950 vs. 550 U/L; *P* < 0.05; Figure 3A and Table 1). This activity was significantly diminished by co-treatment with CB7 (1485 U/L; *P* < 0.05) (Figure 3A and Table 1). Similarly, GST activity in the livers was significantly lower in group 4 than in group 3 (10.34 vs. 12.22 U/L; *p* < 0.05) (Figure 3B and Table 1). CB7 alone did not significantly affect GST activity (Figure 3B and Table 2).

**Figure 4:**
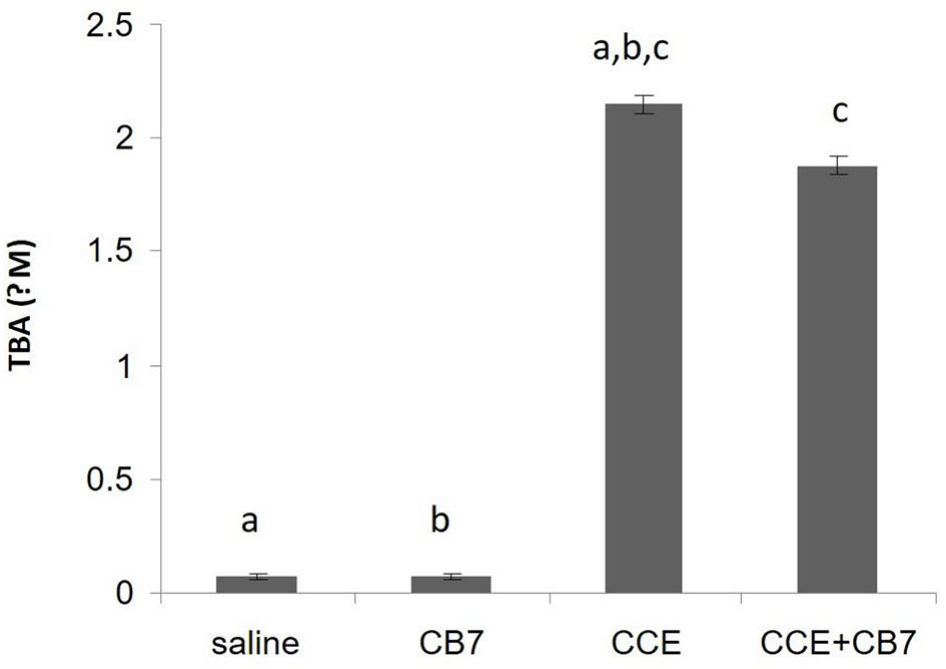
Levels of thiobarbituric acid (TBA), which is indicative of lipid peroxidation, calculated from the livers of mice in each group. Data are presented as mean ± SEM. Bars with similar letters represent statistically significant differences. Ten mice per group (*n* = 10).

We then assessed LPO by measuring the number of TBA-reactive substances. Compared with the control group 1, a marked increase in LPO was observed in group 3 (0.067 vs. 2.152 μM; *P* < 0.001; Figure 4). However, group 4 showed reduced LPO compared with group 3 (1.877 vs. 2.152 μM; *P* < 0.05). Furthermore, LPO was not significantly affected in group 2 compared with the control (0.072 μM; *P* > 0.05).

**Figure 5:**
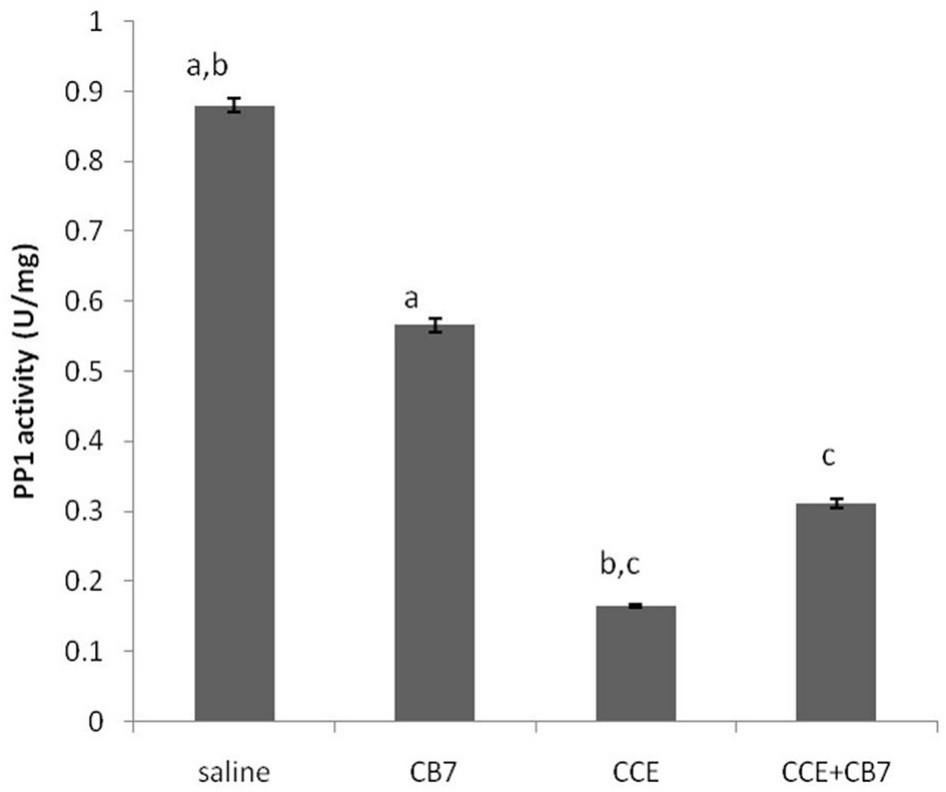
Protein phosphatase-1 (PP1) activity in the livers of mice in each group. Data are presented as mean ± SEM. Bars with similar letters represent statistically significant differences. Ten mice per group (*n* = 10).

Figure 5 and Table 1 present the spectrophotometric determination of PP1 activity for the four groups. The injection of CCE alone (group 3) significantly diminished PP1 activity. This decrease was partially but significantly reversed in group 4. Surprisingly, CB7 alone (group 2) also decreased PP1 activity, though not as markedly as CCE.

The safety and toxicity of CB7 in mice were further tested and validated. The results revealed the absence of any significant changes in various biochemical markers of hepatic function (Table 2).

## Discussion

The present study illustrated that CB7 can serve as a chemoprotectant and can effectively diminish CCE-induced liver damage in mice. This is the first report that demonstrated the protective role of CB7 against CCE-induced liver damage. Our results show that CCE induced liver enlargement resulting from massive intrahepatic hemorrhage, which is consistent with previous findings that MC-LR inhibits PP1, a serine/threonine phosphatase (Honkanen et al., 1990). Inhibition of PP1 is known to increase cytoskeletal protein phosphorylation (Ohta et al., 1992), thereby disrupting the liver cytoskeleton (Hooser et al., 1989, 1991). Our observation that CB7 alone reduced PP1 activity without affecting liver size suggests that the hyperphosphorylation of cytoskeletal proteins was not sufficiently robust to significantly disrupt the integrity of hepatic cells. However, the detailed mechanism of such interactions remains to be investigated, especially considering that the crude extract contains several other components. In fact, the low water solubility of the crude sample precludes a nuclear magnetic resonance measurement that is necessary to determine the site or mode of interactions. It is also notable that CB7 is not entirely soluble in organic solvents (Uzunova et al., 2010). Therefore, regardless of the chemical tool used, the binding patterns can only be studied by monitoring the interaction of CB7 containers with standard MC-LR (Harada et al., 1990).

ALT is a routinely used clinical indicator of hepatocellular injury. The present study illustrated that CB7 protects mice against CCE-induced liver damage by reducing ALT activity upon the coadministration of CB7 and CCE. Similar results were observed for GST, a phase II detoxification enzyme, which was also revealed to detoxify MC-LR (Pflugmacher et al., 1998). Taken together, these observations clearly indicate the protective role of CB7 against CCE-induced liver damage.

The pharmacokinetic profile of CB7 has already been studied both in Balb/c mice and in Sprague Dawley rats after i.p. or i.v. administration, respectively. The i.p. plasma clearance in mice fits a 2-compartment model with exponential decay profile, where clearances of t_1/2_α = 17.0 min and t_1/2_β = 270.7 min are noted. Likewise, in Sprague Dawley rats, i.v. administration appeared to follow a multiphasic clearance model. Bio-distribution of CB7 in mice following i.p. administration show detection in the spleen, liver and brain 10 min post-administration, while activity in the kidneys occurred in two phases, at around 20 and 180 min. Due to this significantly higher activity in the kidneys (compared to that in liver), it was suggested that CB7 is excreted from the kidneys unchanged. It is worth mentioning that low levels of CB7 accumulate in the spleen and liver compared with the plasma and kidneys, while the brain accumulates the least activity, likely indicating that CB7 in unlikely to be able to cross the blood brain barrier (Li F. et al., 2017).

### Conclusion

Based on the biochemical examination of the extracted livers of mice following CCE treatment with or without CB7, CB7 reduced the toxicity of CCE *in vivo*. The use of chemoprotectants against *M. aeruginosa* cyanobacterial toxicity has been the subject of several studies. However, the novelty of the present study lies on the use of a synthetic organic receptor instead of free radical scavengers, Ca^2+^ channel blockers, or enzyme inducers (Hermansky et al., 1991; Atencio et al., 2009). We demonstrated the potential application of CB7 as an adjuvant in toxicological pharmacology.

## Data Availability Statement

The raw data supporting the conclusions of this article will be made available by the authors, without undue reservation.

## Ethics Statement

The animal study was reviewed and approved by Animal house unit of Yarmouk University.

## Author Contributions

NS created the idea and wrote the final draft. NS and SA-J conducted all experimental work. AE and WN evaluated the final draft and added more references. All authors contributed to the article and approved the submitted version.

## Conflict of Interest

The authors declare that the research was conducted in the absence of any commercial or financial relationships that could be construed as a potential conflict of interest.
